# Tangent screen perimetry in the evaluation of visual field defects associated with ptosis and dermatochalasis

**DOI:** 10.1371/journal.pone.0174607

**Published:** 2017-03-29

**Authors:** Molly L. Fuller, César A. Briceño, Christine C. Nelson, Elizabeth A. Bradley

**Affiliations:** 1 Department of Ophthalmology, Wake Forest Baptist Health, Winston-Salem, North Carolina, United States of America; 2 Department of Ophthalmology, Mayo Clinic, Rochester, Minnesota, United States of America; 3 Department of Ophthalmology, Kellogg Eye Center, University of Michigan, Ann Arbor, Michigan, United States of America; Samsung Medical Center, Sungkyunkwan University School of Medicine, REPUBLIC OF KOREA

## Abstract

**Purpose:**

To determine if tangent visual fields gathered during assessment of superior visual field deficits caused by blepharoptosis and dermatochalasis offer good correlation to clinical exam in a time and cost efficient manner.

**Methods:**

Prospective, observational case series. Subjects included all patients referred to a single surgeon (CCN) who underwent surgical correction of blepharoptosis and/or dermatochalasis. Preoperatively and postoperatively, upper margin-to-reflex distances were assessed. Tangent visual fields were performed in a timed fashion and analyzed for degrees of intact vision in the vertical meridian and degrees squared of area under the curve. Data were compared by Student t-tests and Pearson correlation coefficients.

**Results:**

Mean preoperative superior visual fields with the eyelid in the natural position measured 8° in the vertical meridian. Measurements in the vertical meridian and area under the curve showed excellent correlation (r = 0.87). Patients with ptosis showed strong correlation between margin-to-reflex distance and superior visual fields. Patients completed field testing faster than reported times for automated or Goldmann testing. Finally, tangent screens were the least expensive type of equipment to purchase.

**Conclusions:**

Tangent visual fields are a rapid and inexpensive way to test for functional loss of superior visual field in patients with upper eyelid malposition. Our data revealed potential differences between tangent screen results and published results for automated or Goldmann visual field testing which warrants further studies.

## Introduction

Superior visual field testing is a common practice during the evaluation of blepharoptosis and dermatochalasis as it provides objective evidence of functional visual limitation. Many insurance providers require this type of evidence to authorize payment for surgical correction of upper eyelid malposition, categorizing the issue as “functional” rather than “cosmetic.” Medicare’s visual field testing requirements vary by region based on differences prescribed by local coverage determinations. Many carriers mandate superior or lateral visual field limitation of at least 12° or 30% of upper field of vision for surgery to be a covered benefit.[1] Acceptable documentation of visual field impairment includes manual kinetic perimetry by tangent screen or Goldmann perimeter, or static automated perimetry.[1]

Recently, ASOPRS (American Society of Ophthalmic Plastic and Reconstructive Surgeons) released a white paper offering guidelines for the evaluation and testing of patients with blepharoptosis and dermatochalasis.[2] Recommendations were offered to create more uniformity on the part of surgeons and insurers, with reliable, consistent, evidence-based information to support surgical planning. Guidelines include key exam findings of an upper eyelid margin or skin fold at or below 2 millimeters (mm) above the corneal light reflex and decreases in superior visual fields of 12° or 30%. Numerous articles provide evidence that ptosis leads to decreased superior visual fields with greater effects when patients assume a reading or downgaze position [3–10]. Additionally, eyelid malposition and field defects have been consistently correlated with decreased patient functioning and quality of life.[3–5, 11–13] However, nearly all studies describing eyelid related decreases in superior visual fields have been performed by automated perimetry.

Studies have shown that the use of automated visual field testing predominates in the preoperative testing of ptosis and dermatochalasis. In 2011, Aakalu and Setabutr published a survey of American Society of Ophthalmic Plastic and Reconstructive Surgeons members which received a 38% response rate, of whom 54.5% used automated static perimetry, 29.3% used Goldmann perimetry, and 17.2% used tangent screens[14]. However, in a comparison of patient preferences for automated versus Goldmann manual testing, Alniemi, et al, found that manual testing was preferred by patients.[15] Unfortunately, as of 2007, the Goldmann perimeter has been out of production. This leaves clinicians with more limited and potentially more expensive options, including the multifunctional Octopus^®^, when attempting to equip their clinics for visual field testing. Few studies have evaluated the performance of tangent screens in patients with ptosis or dermatochalasis.

Our study was designed to determine if tangent screen testing is a valid, time- and cost-efficient method of testing superior visual fields in patients with ptosis and dermatochalasis. Additionally, because the linear measurement of fields in the vertical meridian is more commonly reported, we sought to determine if the measurement of superior visual field area offered further useful information.

## Methods

Study design: Prospective, observational case series. All patients referred to a single surgeon (CCN) for upper eyelid malposition between January 2012 and June 2014 were invited to enroll in this prospective, observational study if their evaluation led to planned surgical correction by blepharoplasty, ptosis repair, or both. Exclusion criteria included 1) previous or concurrent surgery potentially affecting superior fields (e.g., eyelid wedge resection, brow lift), other than previous uncomplicated blepharoplasty or involutional ptosis repair; 2) ptosis repair by frontalis sling; 3) absent tangent field documentation (see Results section). No patients were excluded based on known or potential ocular comorbidities or visual field defects caused by neuro-ophthalmic, glaucomatous, or retinal disease.

**Fig 1:**
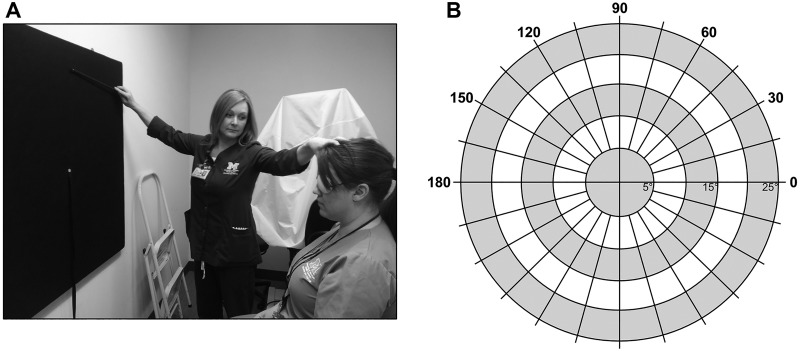
Tangent screen perimetry. A. Patient seated one meter from an eye level tangent screen with the technician holding the target wand against the pattern of isopters and radians. B. Test pattern for the superior visual field shown above the central fixation point.

The study was given exempt status by the University of Michigan Institutional Review Board. Oral informed consent and written HIPAA authorization were obtained prior to enrollment of each participant. Consent was obtained for publication of photography of individuals in Fig 1. Pre- and postoperative clinical evaluation included Snellen visual acuity testing, superior margin-to-reflex distance measurements (MRD1), external photography, and tangent visual field testing.

Visual field testing was completed under standard conditions in a dedicated testing room with ambient indoor lighting.[16] (Fig 1) All tangents were administered by one of a team of experienced oculoplastic and neuro-ophthalmic technicians who regularly provides automated and manual visual field testing and stays with the patient for the entire test. Patients were seated one meter from a tangent screen and instructed to maintain upright posture with the head facing forward, avoiding chin up or chin down position. The non-testing eye was occluded. A target object of a 3 mm white spot on an 18 inch wand was presented on a field with the widest isopter at 25° superior to the horizon and meridians at 15° intervals. Preoperatively, tangent visual fields were performed with the patient’s upper eyelid in its natural position and taped in an elevated position clearing the superior pupillary margin by at least 1 mm to identify the visual field defect due to upper eyelid malposition. The technician would repeat meridians in the event of inconsistent responses and would document a rating of test reliability in the event of difficulty with testing. A timer was used to record the duration of the test for each eye of 30 consecutive patients, based on statistics that n = 30 was an appropriate sample size to offer a good estimate of the true value for the total population. Postoperatively, testing was completed with the eyelid in the natural position only.

Tangent tracings were analyzed in Adobe^®^ Photoshop^®^ to document vision in the superior vertical meridian (Vertical) by degrees and area under the curve (Area) by degrees^2^. Natural, taped, and postoperative field measurements and pre- and postoperative MRD1 were compared by paired t-test. MRD1, Vertical and Area were compared by Pearson correlation coefficient. Statistical analysis was performed in JMP 10^®^ software.

The cost of purchasing basic testing equipment was assessed through online searches (google.com search for “tangent screen” and “tangent perimetry”) and discussions with company sales representatives, selecting the lowest possible cost for each item. Because Goldmann perimeters are no longer manufactured, the cost of a refurbished item available on an internet auction site (eBay.com search for “Goldmann visual field” and “Goldmann perimeter”) was used. Tangent screen kits were priced to include the rigid target field, wand, and target discs. Only automated perimeters capable of performing a Goldmann-style peripheral field assessment were discussed with the Minnesota regional company sales representative of Zeiss (Humphrey 740i) and Haag Streit USA (Octopus 900). Costs of visual field testing equipment were assessed and directly compared.

## Results

### Demographics

A total of 99 eyelids in 52 patients were included in the complete data analysis: blepharoplasty = 61 eyelids (31 patients), ptosis repair = 25 eyelids (15 patients), both = 13 eyelids (7 patients). One patient had blepharoplasty on one side and combined surgery on the contralateral side. There were 51 operated right upper eyelids and 48 operated left upper eyelids. Distance visual acuity in the better seeing eye ranged from 20/20 to 20/30 and in the worse seeing eye ranged from 20/20 to 20/150. Several of the patients with decreased distance vision had surgically or contact lens induced monovision. Eleven additional patients (for a total n = 63) who originally enrolled and had their preoperative fields recorded were excluded for analysis of changes in MRD1 and superior visual fields due to absence of postoperative visual fields. These patients either did not have a field performed due to an oversight during the postoperative clinic visit, because it is not standard clinical practice to retest after surgery, or their results were lost during the rollout of a new electronic medical records system. Their data was included in TVF Times and analysis of correlation between Vertical and Area measurements.

### Visual field analysis

**Fig 2:**
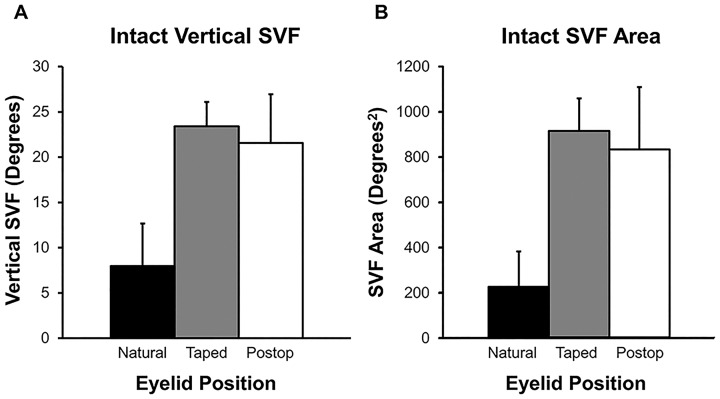
Tangent fields measured in the vertical meridian and area under the curve. Patients had tangent fields analyzed in the vertical meridian (A. Vertical SVF) and area under the curve (B. SVF Area) for their preoperative and postoperative visits. Both preoperative taped and postoperative measures were significantly greater than preoperative in the natural position (p<0.0001). Preoperative field loss calculated as taped position-natural position averaged 15.6° and 697°^2^. Surgery induced a mean field increase of 13.6° and 609°^2^.

First, linear superior fields in the vertical meridian (Vertical), the most commonly used parameter of visual fields, was assessed. The Vertical measure was 8° ± 5° (mean ± SD) in the preoperative natural position, 23° ± 3° in the preoperative taped position, and 22° ± 5° in the postoperative position. Both the taped and postoperative Vertical were significantly greater than the natural preoperative Vertical measure (p<0.0001) (Fig 2). The average increase from the pre to postoperative state was 13.6°.

Next, tangent fields were measured for area under the curve of the tracing (Area). The Area measure was 223°^2^ ± 157°^2^ in the preoperative natural position, 913°^2^ ± 144°^2^ in the preoperative taped position (76% VF loss), and 832°^2^ ± 275°^2^ in the postoperative position (73% VF loss). Both the taped and postoperative Areas were significantly greater than the natural preoperative Area (p<0.0001) (Fig 2). The average increase in Area from the pre to postoperative state was 609°^2^ (273% increase).

**Fig 3:**
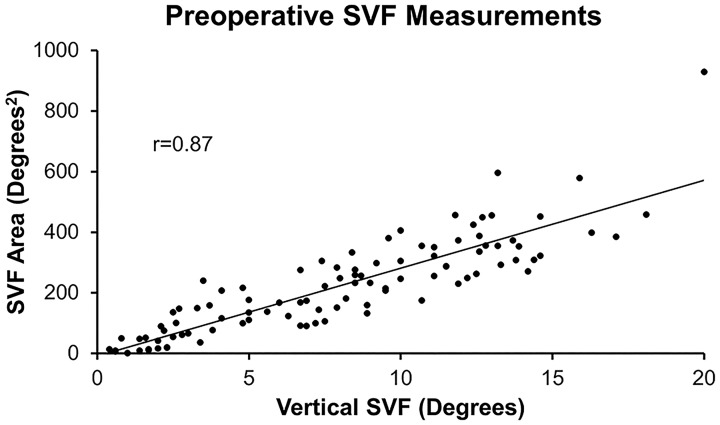
Correlation of Vertical to Area measures. Preoperative measurements were recorded with the eyelids in the natural position. Degrees of superior field in the Vertical meridian were very well correlated with degrees^2^ of Area under the curve on tangent screen (r = 0.87).

Preoperative Vertical and Area measures with eyelids in the natural position were very strongly correlated (r = 0.87) (Fig 3). When examined by surgical subgroup, correlations were nearly identical: blepharoplasty, r = 0.9; ptosis, r = 0.86; both, r = 0.87. Mean values for pre- and postoperative testing in each surgical subgroup is shown in Table 1.

**Table 1:** Measures of clinical exam and visual fields, by surgical subgroups.

	Surgery Group (# of eyelids)							
	Blepharoplasty (61)		Ptosis Repair (25)		Both (13)		Ptosis + Both (38)	
Mean	Preop	Postop	Preop	Postop	Preop	Postop	Preop	Postop
MRD1^a^, millimeters	2.4	3.4	-0.2	2.9	0.4	3.2	0.1	3.1
Vertical SVF^b^, degrees	7.8	20.9	8.1	21.9	8.4	23.9	8.3	22.9
SVF^b^ Area, degrees^2^	223	798	202	843	262	969	232	906

Patients were divided into surgical group. Pre- and postoperative data was compiled, including MRD1 and visual field measurements of the vertical meridian by degrees and superior area by degrees^2^.

^a^: MRD1, superior margin to reflex distance;

^b^: SVF, superior visual field

### Margin-to-reflex distance

**Fig 4:**
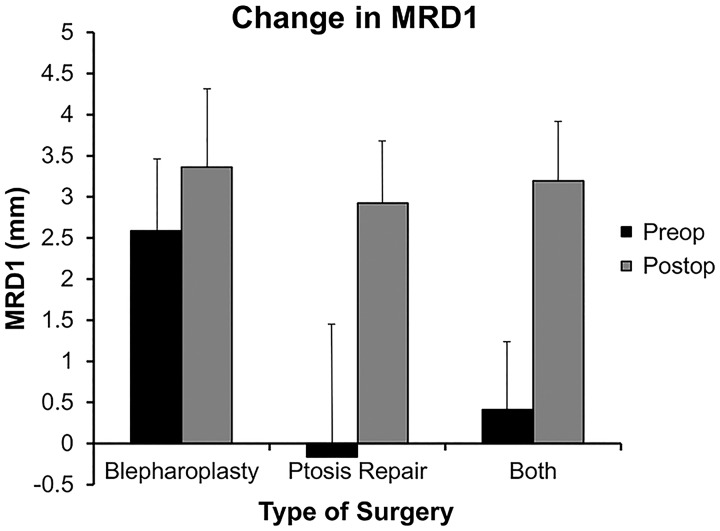
Surgically induced changes in MRD1. Patients were divided into surgical group and the change in MRD1 measurement from preoperative to postoperative visit was determined. Increases were found in all groups: blepharoplasty = 0.8 mm, ptosis repair = 3.1 mm, blepharoplasty and ptosis repair = 2.8 mm (p<0.0001 for all groups).

Absolute measure of patients’ MRD1 was recorded before and after surgery. Patients who underwent blepharoplasty alone had a pre- to postoperative increase in MRD1 from an average of 2.6 mm to 3.4 mm for a mean difference of 0.8 mm (p<0.0001). Patients who underwent ptosis repair alone had a pre- to postoperative increase in MRD1 from an average of -0.2 mm to 2.9 mm for a mean difference of 3.1 mm (p<0.0001). Patients who underwent combined blepharoplasty and ptosis repair had a pre- to postoperative increase in MRD1 from an average of 0.4 mm to 3.2 mm for a mean difference of 2.8 mm (p<0.0001) (Fig 4).

**Fig 5:**
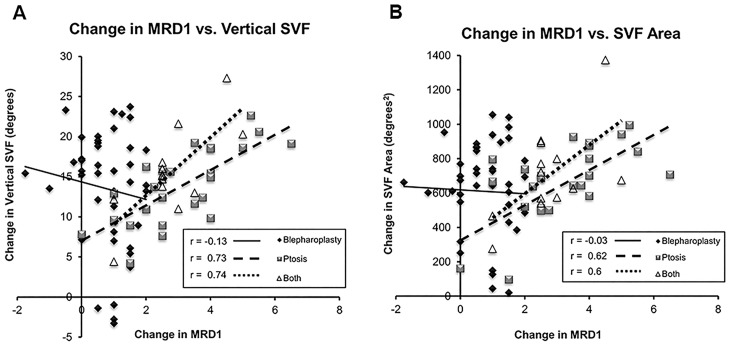
Correlation between MRD1 and superior fields. Patients were divided into surgical group and MRD1 was correlated with Vertical (A) and Area (B) measures. Patients undergoing ptosis repair or combined surgery had an excellent correlation between MRD1 and both measures of superior field. There was limited correlation in the patients who underwent blepharoplasty alone.

Finally, the MRD1 measure was correlated to the tangent perimetry results for Vertical and Area measures. Preoperative MRD1 did not correlate strongly with Vertical (r = 0.2) or Area measures (r = 0.2) in patients who underwent blepharoplasty alone. Preoperative MRD1 did correlate well with Vertical (r = 0.62) and Area measures (r = 0.62) in patients who underwent ptosis repair alone. In patients who underwent combination blepharoplasty and ptosis repair, preoperative MRD1 correlated with Vertical (r = 0.65) better than with Area measures (r = 0.48). Fig 5 shows the correlation between the changes in MRD1 and Vertical or Area measures divided into surgical groups.

### Test duration

Mean time for unilateral tangent testing was 3:11 (minutes:seconds; 95% CI = 2:07–4:15) and bilateral tangent testing was 6:23 (95% CI = 5:26–7:20). Median time for unilateral testing was 2:31 and for bilateral testing was 5:04. These times include the assessment of visual fields with the eyelids in both the natural and taped positions.

### Equipment costs and reimbursement

Tangent screen sets like the one in Fig 1 are the least expensive visual field equipment to obtain at $250 (Richmond Products, Good-Lite Company, New Mexico, USA). Of note, even more affordable sets are available as a roller shade rather than rigid target field or with meridians every 22.5° rather than every 15°. Refurbished Goldmann equipment is potentially available for $300 to $3000. Given the transient nature of online auction sales, there is variation in product cost and availability over time, but a recent search found a Haag Streit Goldmann perimeter with an asking price of $500.[17] The purchase of an automated perimeter is many times more expensive, starting at $13,000 for the Humphrey VF Analyzer 740i or $26,000 for a multifunctional analyzer with automated kinetic testing options, the Haag Streit Octopus 900. These prices may vary based on local markets. Medicare reimbursement for these tests most commonly falls within billing code 92081, described as “limited visual field examination (e.g. tangent screen, Autoplot, arc perimeter, or single stimulus level automated test, such as Octopus 3 or 7 or equivalent),” regardless of the perimeter used.[18]

## Discussion

Our population of patients with ptosis and dermatochalasis had decreases in superior visual fields which could be documented by tangent testing in both the vertical meridian and superior area of vision. We established that there is an excellent correlation between these two measurements, and both show improvement after ptosis repair, blepharoplasty, or both. The time taken to perform this testing was substantially less than reported times for Goldmann or automated testing.[15] To our knowledge, this report is the first large series of patients to have eyelid malposition evaluated by tangent screen, and thus offers new insight into the relationship between eyelid position and visual fields.

Documentation of a decrease in intact superior visual fields is required to support the functional nature of blepharoptosis or dermatochalasis, distinguishing these conditions from cosmetic concerns.[1] Over the past several decades, third party payors have instituted a requirement for the superior field to be decreased by 12° or, often, 30%. The relationship between upper eyelid malposition and loss of superior visual field has been examined many times using Goldmann or automated testing. In 2011, The American Academy of Ophthalmology released an Ophthalmic Technology Assessment to evaluate the literature for preoperative indications for and surgical results of blepharoplasty and blepharoptosis repair.[13] Numerous indicators were identified including superior visual field restriction of 12° or 24%, but nearly all studies included in the review used Goldmann or automated testing. The ASOPRS white paper released last year makes similar recommendations and states that acceptable forms of testing include tangent screen, Goldmann or automated testing, but there is little literature to support the idea that these tests offer comparable or interchangeable output.

Our data reveal that tangent testing identifies more limited superior visual fields for the same MRD1, compared with other published data. Correlation between MRD1 and superior field restriction has been well documented with Goldmann and automated testing.[11],[3],[12] In Federici et al, where ptosis was assessed by Humphrey or Goldmann testing with a III4e target, the more ptotic eyelid showed a mean preoperative MRD1 of 0.1 mm and a mean superior visual field height of 19°, and the less ptotic eyelid showed a mean preoperative MRD1 of 1.6 mm and a mean superior visual field height of 31°.[5] When the eyelid data were combined, the mean preoperative MRD1 was 1.3 mm and superior visual field height was 28° with a correlation of r = 0.42. Meyer, et al, 1993 used threshold automated fields to test ptosis induced by placement of an external gold weight on the eyelids of 20 healthy volunteers.[4] In the subjects with moderately-severe ptosis, the mean calculated MRD1 was -0.1 mm and the superior field height encompassing sensitivities greater than 10 dB was 26°. In contrast, our study identified that patients with ptosis had a mean preoperative MRD1 of 0.1 mm and superior field height of 8°, showing a strong correlation of r = 0.62 (without blepharoplasty) and 0.65 (with blepharoplasty) between the two measurements.

Our data also show that the area of superior field is a useful measure. The vertical field measured at the 90° meridian is more easily calculated than an absolute or percent change in area across the superior hemifield. Additionally, patients may have variation in eyelid contour with greater ptosis or dermatochalasis affecting the nasal or temporal portion of the visual field which may not be captured at the vertical meridian. Therefore, we sought to determine if the Vertical measure acted as an appropriate surrogate for Area. Indeed, the correlation between the preoperative Vertical and Area measures was excellent. Additionally, both measures had pre to postoperative surgical changes that correlated to expected surgical changes in MRD1. As expected, patients undergoing blepharoplasty alone had the weakest correlation with MRD1, whereas patient undergoing ptosis repair had strong correlations with MRD1, though in the patients undergoing combined surgery, the MRD1 more strongly correlated with the Vertical than Area measure. This may reflect the variable influence of temporal hooding in dermatochalasis. Maamari, et al, recently published that oculoplastic surgeons underestimate the area of a visual field defect, while a custom designed iPhone^®^ (Apple, Inc., Cupertino, CA) application based on ImageJ software was extremely accurate.[19] This is of particular importance for interpreting tangent results which detected smaller areas of intact vision preoperatively. The consequence of smaller preoperative fields is that the percentage of area gained by eyelid elevation with tape (76%) or surgery (73%) was much greater than the 30% required by third party insurers. Therefore, by tangent testing, more patients would qualify as functionally reduced by area than by vertical measurement. In the future, integration of visual field images directly into the electronic medical record could allow for automatic calculations to be generated and documented with coordinated software, making the use of area measurements easier.

The reason for detecting smaller visual fields by tangent testing is likely multifactorial. First, tangent screens create a ceiling effect. The largest isopter tested by our tangent screen is at 25° from central fixation. Due to the request by third party payors for patients to have a 12° decrease in vertical fields to qualify for surgical reimbursement, our patients underwent surgery and were enrolled in the study only when preoperative field limitations were advanced. The second likely reason that tangent fields are smaller is retinal sensitivity to the testing stimulus. Riemann, et al, compared Goldmann and automated fields in patients with ptosis.[20] Their study found preoperative vertical fields with eyelids in the natural position to be 28° by Goldmann and 24° by automated testing. Their Goldmann data was gathered with a V-4-e spot size (diameter = 9.03 mm), which gave larger fields than the more standard III-4-e spot size (diameter = 2.26 mm) which they also tested. But both the automated and Goldmann perimeters use light-emitting target objects of relatively higher luminance than the reflected ambient lighting provided by the 3 mm tangent target object. Given the various testing formats, it is not possible to directly compare the test objects from different perimeters, but it is reasonable to speculate that the tangent target would lead to smaller test fields than either illuminated Goldmann or automated target. In fact, a case reported by Cahill, et al, showed a patient with ptosis causing superior field defects to within 20° and 25° in each eye on tangent screen, but upon testing by Goldmann perimetry, the defects were identified at approximately 30° and 40°.[11] Further direct comparisons between these exams have never been completed but would make an excellent future study.

Other important considerations for the use of different perimeters are cost and logistics. Goldmann perimeters are no long available commercially, thereby limiting the testing options for the formal evaluation of fields. New automated perimeters are available with combined capabilities of static and kinetic perimetry, but this equipment is quite expensive and manual perimetry is favored by patients.[15] Our report shows that tangent screens carry low direct and indirect costs. Comparisons of estimated equipment purchasing costs are widely divergent, with the cost of tangent screen equipment being so low as to be completely covered by the reimbursement of just a handful of tested patients. Some practices already have automated equipment which is used for testing of other ophthalmic conditions. Use of this equipment for ptosis and dermatochalasis evaluation is reasonable, but in many busy referral centers, such equipment is in high demand. There may be wait times for either the perimeter or a technician to perform the test, so the ability of a tangent screen to offer quick, effective testing is extremely valuable. Other surgeons opt to have their patients referred to them with visual fields completed by a referring provider. Again, this is a reasonable approach, but this negates the ability of the surgeon to charge for the technical fee for the testing, and thus represents lost income.

As for indirect costs, if we assume no change to technician staffing between test modalities—that the technician remains in attendance with the patient during all types of visual field testing to encourage proper patient positioning, fixation, and attention—then the time saved during tangent testing is also money saved in employee efficiency. We can to compare our test times with the data from Alniemi, et al, as their report clearly states that test times encompassed testing of superior fields of both eyes in the ptotic and non-ptotic positions, just as our study did. In their study, testing by Goldmann perimetry took 12:10 while automated perimetry took 18:50, compared to our tangent testing time of 6:23. Other studies have reported testing times for visual fields, such Ho, et al, who used custom designed automated ptosis fields taking an average of 4.18 minutes preoperatively, but without testing of the additional elevated eyelid position. Furthermore, it was not specified if testing was unilateral or bilateral.[6] Patipa tested eight ptosis patients by automated fields and found a range of 3.35–6.12 minutes with a mean of 5.32 minutes for unilateral testing.[10] Our tangent test times were very efficient compared to the literature.

While the majority of patients had successful eyelid correction achieving postoperative MRD1 at or above 3 mm without overhanging eyelid skin, several patients experienced less than ideal improvement in fields with Vertical and Area measures more similar to preoperative natural position than taped position. In reviewing these cases, we found surgical failures including patients with postoperative MRD1 less than 2.5 mm (6 eyelids), patients with a Hering’s drop in eyelids which were the preoperatively less ptotic side (3 eyelids), and patients with excellent eyelid position who had significant brow ptosis causing superior field defects (5 eyelids). Many of these patients later had further surgery to correct the eyelid or brow position. Of note, postoperative visual field results had more variation and a lower mean visual field size compared to preoperative taped values, as can be seen in Fig 2. This reveals the fact that even “successful” surgery is not always able to achieve an artificial ideal.

Our study has several important potential limitations. Our study only enrolled patients who met criteria for insurance coverage, so we cannot comment on the sensitivity or specificity of tangent testing. It is not clear how many patients complained of limited superior visual fields, had MRD1 measurements of 2 mm or less, but did not have “functionally limited” fields on tangent testing per payor criteria. Additionally, our patients underwent each condition of testing only once, so we cannot comment on the reproducibility of the test. Given the similarities with manual administration of a Goldmann visual field, reproducibity may be similar. A direct comparison of tangent fields, Goldmann fields, and Humphrey fields would be enlightening. These would be important issues to assess in future studies.

Rather than continuing to allow third party payors to dictate a narrow set of criteria for functional visual field loss, we should continue to gather data to support the various ways in which eyelid malposition affects patients. In the current reimbursement climate, the burden of proof for the physician to provide evidence of visual field limitation has increased but the reimbursement for blepharoplasty and blepharoptosis repair has declined. Providers need to decide the most time and cost effective way of managing patients while maintaining high patient satisfaction. In this study, we document that tangent visual fields are a time efficient and inexpensive method of testing superior visual fields for ptosis and dermatochalasis. Tangent testing gathers data consistent with the clinical exam, though not identical to published Goldmann or automated testing output. These differences should be considered when formal testing guidelines are recommended.

## Supporting information

S1 Supporting information Excel file of source data and statistical analysis. (XLSX)
